# Insights into the role of neutrophils in neuropsychiatric systemic lupus erythematosus: Current understanding and future directions

**DOI:** 10.3389/fimmu.2022.957303

**Published:** 2022-08-09

**Authors:** Tao Ming Sim, Anselm Mak, Sen Hee Tay

**Affiliations:** ^1^ Department of Medicine, Yong Loo Lin School of Medicine, National University of Singapore, Singapore, Singapore; ^2^ Division of Rheumatology, Department of Medicine, National University Hospital, Singapore, Singapore

**Keywords:** neutrophils, neuropsychiatric systemic lupus erythematosus, extracellular traps, bloodbrain barrier, systemic lupus erythematosus

## Abstract

Central nervous system (CNS) involvement of systemic lupus erythematosus (SLE), termed neuropsychiatric SLE (NPSLE), is a major and debilitating manifestation of the disease. While patients with SLE mostly complain of common neuropsychological symptoms such headache and mild mood disorders that may not even be technically attributed to SLE, many SLE patients present with life-threatening NPSLE syndromes such as cerebrovascular disease, seizures and psychosis that are equally challenging in terms of early diagnosis and therapy. While we are just beginning to unravel some mysteries behind the immunologic basis of NPSLE, advancements in the mechanistic understanding of the complex pathogenic processes of NPSLE have been emerging through recent murine and human studies. The pathogenic pathways implicated in NPSLE are multifarious and various immune effectors such as cell-mediated inflammation, autoantibodies and cytokines including type I interferons have been found to act in concert with the disruption of the blood-brain barrier (BBB) and other neurovascular interfaces. Beyond antimicrobial functions, neutrophils are emerging as decision-shapers during innate and adaptive immune responses. Activated neutrophils have been recognized to be involved in ischemic and infective processes in the CNS by releasing neutrophil extracellular traps (NETs), matrix metalloproteinase-9 and proinflammatory cytokines. In the context of NPSLE, these mechanisms contribute to BBB disruption, neuroinflammation and externalization of modified proteins on NETs that serve as autoantigens. Neutrophils that sediment within the peripheral blood mononuclear cell fraction after density centrifugation of blood are generally defined as low-density neutrophils (LDNs) or low-density granulocytes. LDNs are a proinflammatory subset of neutrophils that are increased with SLE disease activity and are primed to undergo NETosis and release cytokines such as interferon-α and tumor necrosis factor. This review discusses the immunopathogenesis of NPSLE with a focus on neutrophils as a core mediator of the disease and potential target for translational research in NPSLE.

## Introduction

Systemic lupus erythematosus (SLE) is a debilitating autoimmune disease characterized by a myriad of complex and heterogenous clinical manifestations, involving various organs such as the kidneys, skin and the central nervous system (CNS) (1). There is marked unpredictability in the disease course of SLE, often resulting in remissions and flares that lead to cumulative organ damage and mortality (2). The past 5 decades have seen increases in survival rates of SLE patients, though further improvements in survival have been hindered by organ damage to renal and neuropsychiatric systems (2).

Central nervous system involvement of SLE, termed neuropsychiatric SLE (NPSLE), is a major and debilitating manifestation of SLE which often masquerades as common and non-specific neuropsychological features such as headache and mild mood disorders. Such diverse, protean and often non-specific manifestations of NPSLE have made the clinical diagnosis and management of the condition challenging. Given this variability in disease phenotype, robust epidemiology studies are sorely lacking owing to the lack of consistency in inclusion criteria and case definition of neuropsychiatric (NP) events (3). While the true prevalence of NPSLE is unclear, CNS disease has been conservatively estimated to affect more than 20% of SLE patients (4). NPSLE is one of the most complex and challenging facets of SLE and can be mild or severe, focal or diffuse, acute or chronic, all posing a significant negative impact on quality of life (5) and has been found to be associated with an increase in mortality rate of as high as threefold as compared with SLE patients without NPSLE (6).

Research to elucidate the complex pathogenesis of NPSLE has seen recent advancements in mechanistic understanding and we are just beginning to unravel the mysteries behind the immunologic basis of NPSLE. The pathogenic pathways implicated in NPSLE are multifarious and various immune effectors such as cell-mediated inflammation, autoantibodies and cytokines including type I interferons (IFNs) have been found to act in concert with the disruption of the blood-brain barrier (BBB) and other neurovascular interfaces. Current management options for NPSLE are still inadequately optimized due to the lack of treatment targeting NPSLE-specific pathways. Nonetheless, advances in investigational techniques including lupus animal models and advances in neuroimaging have accelerated and augmented the study of NPSLE (7, 8) The aberrant adaptive immune response with loss of self-tolerance with activation of T and B cells leading to the production of pathogenic autoantibodies and systemic inflammation has long been advocated as one of the major mechanisms of the pathogenesis of SLE (9). The contribution of the innate immune system to SLE immunopathogenesis is yet less well understood.

Neutrophils are the most abundant leukocytes and are pivotal effector cells in the innate arm of the immune system, functioning as circulating phagocytes recruited to sites of infection to aid in the clearance of extracellular pathogens (10). Such effector functions of neutrophils are enabled by the presence of adhesion molecules such as selectins and integrins which enable interaction with the endothelium, allowing for chemotaxis, extravasation and migration into inflamed tissues. Neutrophils also display a range of pattern-recognition receptors and complement receptors which are critical in driving phagocytosis (11). Following phagocytosis, the fusion of the phagosome with neutrophil granules and the formation toxic reactive oxygen species enables the killing of engulfed pathogens. Neutrophils are also able to degrade virulence factors and kill bacteria through a unique cellular process involving the release of chromatin and granules as extracellular fibers called neutrophil extracellular traps (NETs) (12). While previously envisaged as a subset of non-specific leukocytes in the front line of immune defense, abnormalities of neutrophils have been found to be associated with the development of various autoimmune diseases such as SLE (13), rheumatoid arthritis and anti-neutrophil cytoplasmic antibody (ANCA)-associated vasculitis (14), making neutrophils increasingly recognized as more sophisticated cells of vital importance in the maintenance of immune homeostasis (15).

An overarching theme in the discussion of neuroimmune interactions are the unique properties of the brain vasculature and parenchyma. The CNS is an immune privileged site owing to the presence of the selective and restrictive BBB that limits exposure of the CNS to various toxins, pathogens, systemic immune mediators and cellular elements (16). Over the years, novel routes that enable the disruption of the BBB and consequent leukocyte infiltration to the CNS have been described (17–19). Disruption of the BBB also allows for other serum-borne immune effectors such as autoantibodies to cross into the CNS. Autoantibodies against a wide array of cellular antigens have been described in SLE and in particular, anti-neuronal antibodies have been found to be associated with NPSLE (20). Disruption of the BBB and increased penetrance of leukocytes and autoantibodies work in tandem to contribute to neuroinflammation in NPSLE.

This review discusses the current understanding of the pathogenic roles of neutrophils in SLE, proposing unifying mechanisms through which these intricate immune cells operate and contribute to the development of NPSLE. This review will also highlight the potential for translational research in NPSLE including therapies targeted at neutrophils and associated pathways.

## Neutrophils in SLE

### Isolation of low-density neutrophils in SLE

In 1986, Hacbarth and Kajacsy-Balla were the first to discover low-density neutrophils (LDNs) or low-density granulocytes in the peripheral blood mononuclear cell (PBMC) preparations of adult SLE patients and found that the presence of this low buoyancy subset of neutrophils was correlated to SLE disease activity (21). They suggested that humoral factors in the plasma were responsible for *in vitro* activation of neutrophils, causing cellular degranulation and the formation of this unique phenotype of neutrophils in SLE patients. More recent studies of the nuclear morphology by transmission electron microscopy suggest that the low-density phenotype is due to LDNs being band or immature forms of neutrophils (22).

With the advent of transcriptome profiling, Bennett et al. performed microarray analysis of PBMCs isolated by density gradient in paediatric SLE patients and identified high expression of neutrophil-specific genes which was coined the ‘granulocyte signature’ (23). This ‘granulocyte signature’ was found to be associated with the increase in number of low-density neutrophils (LDNs) in the PBMC. These observations were echoed by another investigator who demonstrated that gene arrays performed on low-density cells isolated by Ficoll-Histopaque gradient centrifugation of bone marrow aspirates from SLE patients showed upregulation of granulopoiesis-related genes found in early stages of neutrophil development (24).

LDNs possess altered function as compared to normal neutrophils. LDNs exhibit enhanced capacity to synthesize cytokines including type I IFNs, decreased ability to phagocytize pathogens, and a marked propensity to spontaneously form NETs (25, 26). This altered physiology of LDNs is suggested to be responsible for autoimmunity and inflammation in SLE. NETs, which comprise chromatin fibers, histones and granular proteins, act as externalized autoantigens that activate the adaptive immune system and contribute to the pathogenesis of SLE (25). NETs synthesized by LDNs display increased externalization of immunostimulatory proteins such as interleukin-17 (IL-17), a vital cytokine associated with T cell activation, especially in chronic inflammation and autoimmunity (26, 27). The tendency of LDNs to spontaneously form NETs is thought to be due to an increase in the release of mitochondrial reactive oxygen species (28, 29). Oxidized mitochondrial DNA derived from LDN NETs is pro-inflammatory and is a potent stimulator of IFN genes, in particular it is able to induce type I IFN signaling in target cells (28). Mitochondrial DNA, unlike genomic DNA, contains hypomethylated CpG similar to bacterial DNA (30). Specifically, oxidized mitochondrial DNA in the form of NETs signals through the DNA sensor STING but *via* TLR9 in the form of extruded nucleoids from SLE neutrophils to mediate type I IFN activity (28, 30). Hence, the interferogenic capacity of oxidized mitochondrial DNA is clearly context dependent. In addition, NETs have also been found to cause endothelial damage and exposure of immunostimulatory molecules, further compounding the inflammatory reaction (26, 31).

LDNs have also been found to exhibit a distinct cytokine profile involving increased secretion of tumor necrosis factor alpha (TNF-α), IFN-γ and type I IFNs which are implicated in SLE pathogenesis (32). LDNs are also reported to express higher levels of TNF-α than high-density neutrophils (25). Dysregulation of type I IFN signature is well-characterized in SLE (33) and was previously attributed to increased type I IFN secretion by plasmacytoid dendritic cells (pDC) (34). Recent work have shown that neutrophils have the capacity to secrete type I IFN upon specific types of stimulation including granulocyte-colony stimulation factor and polyinosinic:polycytidylic acid (35, 36). *In vitro*, LDNs have been reported to synthesize type I IFNs in sufficient amounts to interfere with the differentiation of endothelial progenitor cells into mature endothelium (25, 31), a phenomenon observed in SLE and implicated in the development of early onset atherosclerosis (37). Interestingly, depletion of pDCs was not found to affect endothelial cell differentiation, suggesting that LDNs may contribute to endothelial disruption, mediated in part by a type I IFN effect (25).

## The role of neutrophils in NPSLE

### NETosis as a source of autoantibody formation

Low-density neutrophils display aberrant properties from normal high-density neutrophils and result in exuberant NETosis and impaired clearance of NETs, providing a mechanism for stimulation of autoimmunity as the nuclear DNA, histones, and granule proteins released in the NETs serve as self-antigens (38).

Aberrant NETosis may explain the longstanding recognition of increased levels of circulating DNA in SLE patients (39). It has also been found that approximately 36% of SLE sera degrade NETs poorly, with inhibitors of DNase I detectable in some patients, while others displayed autoantibodies that coated NETs and mechanically protected against degradation (40). The significance of poor NET degradation is a potentially higher titer of anti-double-stranded DNA antibodies, and correspondingly more complement activation and end-organ damage (41). NETs have also been found to stimulate pDCs to release type I IFNs (42, 43), which lowers the threshold for autoreactivity of both antigen-presenting and antibody-producing cells (44). Further, SLE patients are shown to have autoantibodies to both the self-DNA and antimicrobial peptides in NETs, indicating that NETs serve as a source of novel intracellular autoantigens for subsequent B cell activation and production of autoantibodies (44). This phenomenon may be due to the fact that NETs display increased externalization of immunostimulatory proteins and autoantigens including double-stranded DNA, LL-37 and IL-17 (26).

The formation of NETs is a key mechanism in enabling neutrophils to control infection as NETs are a source of concentrated immune effectors including neutrophil elastase (NE), myeloperoxidase, cathepsin G and proteinase 3 which display microbicidal properties (45, 46). These released immune effectors in NETs are toxic to tissue and have been found to cause apoptosis in endothelial cells and contribute to glomerular injury in lupus nephritis (47, 48). Intrathecal NETosis may contribute to direct neuronal damage in NPSLE, which has been found to contribute to the pathogenesis of memory deficit in Alzheimer’s disease (49, 50). NE and cathepsin G found in NETs have the capacity to potently process and activate cytokines including IL-1α and IL-36 which promote inflammation (51) and may account for indirect damage to the CNS in NPSLE. There is also suggestion that NETs may be a source of vascular and organ damage in SLE as netting neutrophils have been shown to have capacity to cause endothelial damage and infiltrate organs such as skin and kidney (26). A murine study found that circulating neutrophils were able to be primed by antiphospholipid antibodies and promote thrombosis (52).

### Matrix metalloproteinase-9 and neutrophil gelatinase-associated lipocalin

Matrix metalloproteinases (MMPs) are members of the metzincin group of proteases with a wide substrate spectrum which share the conserved zinc-binding motif in their catalytic active site (53). In biological systems, MMPs are zinc- and calcium-dependent endoproteinases that are crucial in degradation and remodelling of the extracellular matrix (ECM) and regulating extracellular tissue signaling networks (54, 55). MMPs are secreted as zymogens and are subsequently activated extracellularly by proteinase-facilitated or non-proteolytic mechanisms (56). MMP expression is closely regulated at the level of gene transcription, secretion, activation and inhibition by tissue inhibitors of metalloproteinases (TIMP) (56, 57).

Matrix metalloproteinase-9, also known as gelatinase B, is one of the most complex MMPs in the human proteasome and proteomic studies have identified possible substrates for MMP-9 that may reach a few hundred (58). MMP-9, which is found to be concentrated in the cerebellum, hippocampus and cerebral cortex of the brain, is responsible for degradation of ECM components including gelatine and type IV, V, XI and XVI collagens during tissue remodelling, which is postulated to influence the disruption of the BBB and neuroinflammation (58, 59). MMP-9 is secreted by a wide array of cells, including neutrophils, macrophages, lymphocytes and fibroblasts, with neutrophils being one of the richest sources of active MMP-9 (60, 61). This is attributed to neutrophils that contain various proteases such as serine proteases (elastase, proteinase 3 and cathepsin G) and urokinase plasminogen activator which promote MMP-9 activation (62).

The relationship between serum MMP-9 levels and SLE disease activity have been studied but results were inconsistent (63–66), possibly due to discrepancies in laboratory techniques employed or epigenetic polymorphisms (66). On the other hand, serum and CSF levels of MMP-9 have been found to be consistently elevated in SLE patients with NP involvement as compared to patients without (67). A study involving 123 patients with SLE who were evaluated clinically, with magnetic resonance imaging (MRI) and CSF analyses of MMP-9 found significant correlation between MMP-9 and intrathecal levels of tau and glial fibrillary acid protein, markers of neuronal degradation, suggesting ongoing neurodegeneration in the brains of NPSLE patients with high expression of MMP-9 (68). This study also found that intrathecal titers of IL-6 and IL-8 correlated with MMP-9 levels in the CSF of NPSLE patients. IL-6 and IL-8 are cytokines demonstrated to have elevated CSF levels in NPSLE patients (69, 70). These proinflammatory cytokines have been shown to stimulate the production and release of MMP-9 in other immune cells including macrophages (71–73). Taken together, a proinflammatory milieu conferred by proinflammatory cytokines such as IL-6 and IL-8 induces MMP-9 production, leading to the neurodegeneration and increased neuronal and astrocytic degradation productions observed in NPSLE (74, 75). IL-8 is also postulated to promote transmigration of activated inflammatory cells across the endothelium, amplifying the effects of ECM and BBB disruption by MMP-9 (68).

One study found that elevated levels of MMP-9 in patients with SLE were associated with MRI indices of cerebral infarcts and NP involvement, particularly cognitive dysfunction (64). Prior studies of giant cell arteritis elucidated that increased MMP-9 expression contributed to leukocyte migration into the vessel wall *via* damage to the internal elastic lamina of the vessels and inflammation (76). In the context of NPSLE, it is postulated that MMP-9 contributes to small-vessel vasculopathy *via* a similar mechanism, causing foci of cerebral ischaemia and infarcts derived from atherosclerosis in cerebral vessels, accounting for cognitive dysfunction (64). MMP-9 has also been shown to contribute directly to neuronal apoptosis and damage to brain parenchyma (77–80), through degradation of laminin which induces apoptosis and transient focal cerebral ischaemia (81).

Neutrophil gelatinase-associated lipocalin (NGAL) is a 25 kDa protein under the lipocalin superfamily. While NGAL was initially found in activated neutrophils, many other cell types including kidney tubular cells also express NGAL in response to noxious stimulants such as ischaemia and inflammation (82). NGAL is completely absent in normal brains but can be induced in the choroid plexus following infectious and inflammatory insults. NGAL potentiates the ability of MMP-9 to increase the permeability of the BBB, and NGAL levels correlate with microglia activation (83). In a prospective study of 60 children with SLE, increase in serum NGAL level was found to be associated with decline in psychomotor speed during an 18-month observation period (84). Nevertheless, the source of NGAL in this cohort was not ascertained. A study in a murine lupus model found that in the CNS, NGAL expression was increased more than 30-fold in lupus mice than normal mice and that NGAL promoted damage through mechanisms established to be linked to NPSLE such as disruption of the BBB and direct neurotoxicity (85). To establish the relevance of NGAL to human disease, the authors measured NGAL concentrations in the CSF of patients with NPSLE and healthy controls. It was found that CSF NGAL levels were significantly elevated in NPSLE, raising the potential for future clinical application of measuring NGAL levels in the CSF as a diagnostic biomarker for NPSLE.

## Disruption of the blood-brain barrier

In SLE, end-organ inflammation and damage mediated by immune-complex activation of complement cascade is well-described especially for lupus nephritis (86). However, the understanding of NPSLE is complicated by the presence of the BBB which serves as a physical and biochemical barrier of the CNS through its tight, adherens and gap junctions, specialized structures resulting from the unique interaction of cerebral endothelial cells, pericytes and astrocytic foot processes (87, 88). The CNS is an immune-privileged anatomic site due to the presence of this tightly regulated and highly restrictive BBB which serves as a functional and mechanical barrier, thereby preventing passive transfer of most immune cells, immune complexes, pathological antibodies and mediators including cytokines from the circulation to the CNS (16). BBB disruption is central to the altered homeostasis seen in a myriad of CNS disorders including neurodegenerative conditions such as Alzheimer’s disease and Parkinson’s disease and autoimmune conditions including multiple sclerosis and neuromyelitis optica (89, 90). Therefore, the question central to the study of SLE and its CNS manifestations is whether a breach of BBB integrity is implicated in NPSLE and if so, what the underlying mechanisms are.

Post-mortem autopsy studies of NPSLE brains have shown that histopathological lesions in NPSLE represent a continuum that ranges from non-specific lesions such as focal vasculopathy, to more specific lesions including C4d-C5b complement-associated microthrombi and diffuse vasculopathy (91). The presence of complement deposition is suggested to be a key factor in the interaction between circulating autoantibodies and the thrombo-ischaemic vascular lesions observed in NPSLE. Histopathology showing vasculitis with inflammatory infiltrates of predominantly mononuclear cells and depositions of immunoglobulin and complement, accompanied by fibrinoid necrosis have also been described in NPSLE (92).

The earliest reports of BBB impairment in NPSLE was by Winfield et al. in 1983 (93). Much of what is believed about the loss of BBB integrity in NPSLE is based on evidence from surrogate markers of leakage of serum proteins into the CNS such as Qalb (94, 95). However, lumbar puncture is required to obtain CSF which is an invasive procedure with associated risks, so its application has been relatively limited in the context of clinical diagnosis of NPSLE (96). BBB disruption can be inferred from regions of edema using computed tomography and MRI but these techniques lack spatial resolution to detect small leaks across the BBB (97). With the advent of advanced neuroimaging technique, various methods have been used to assess BBB permeability in SLE patients. They include non-gadolinium-based arterial spin labelled combined with diffusion-weighted imaging for paediatric patients and the gold standard, gadolinium-based dynamic contrast-enhanced MRI in adults (98–101).

Increased BBB permeability has been described in specific structures (e.g. right insular, Brodmann’s area 19,28,36 and 37) and the whole brain in SLE patients (101–103). Of note, increased BBB permeability in the right insular and Brodmann’s area 19 have been associated with anxiety/depression and worse psychomotor speed, respectively (102). In addition, extensive BBB leakage (affecting more than 9% of the brain volume) was associated with lower global cognitive scores and impairment in one or more cognitive tasks (101).

A seminal study involving *ex vivo* analyses of autopsied human brain after haemorrhagic conversion of an acute ischaemic stroke found a strong infiltration of infarcted brain by MMP-9-expressing neutrophils which were associated closely with regions with pronounced basal lamina collagen IV degradation and BBB breakdown (104). Depletion of neutrophils in a rat model of intracerebral hemorrhage displayed muted expression of MMP-9 and reduced neuroinflammation, BBB breakdown and axonal injury (61). A study of cerebrospinal fluid (CSF) and serum levels of active MMP-9 in patients with the multiple sclerosis found elevated levels of MMP-9 which correlated with disease activity and in particular, the MMP-9/TIMP ratio was found to be pivotal in modulating inflammatory disease activity (105). Taken together, this is suggestive that MMP-9-expressing neutrophils, not compensated by an adequate down-regulatory effect of TIMP, can facilitate BBB breakdown through disruption of the ECM and basal lamina, possibly potentiating invasion of the CNS by other immune cells and perpetuating neuroinflammation.

**Figure 1:**
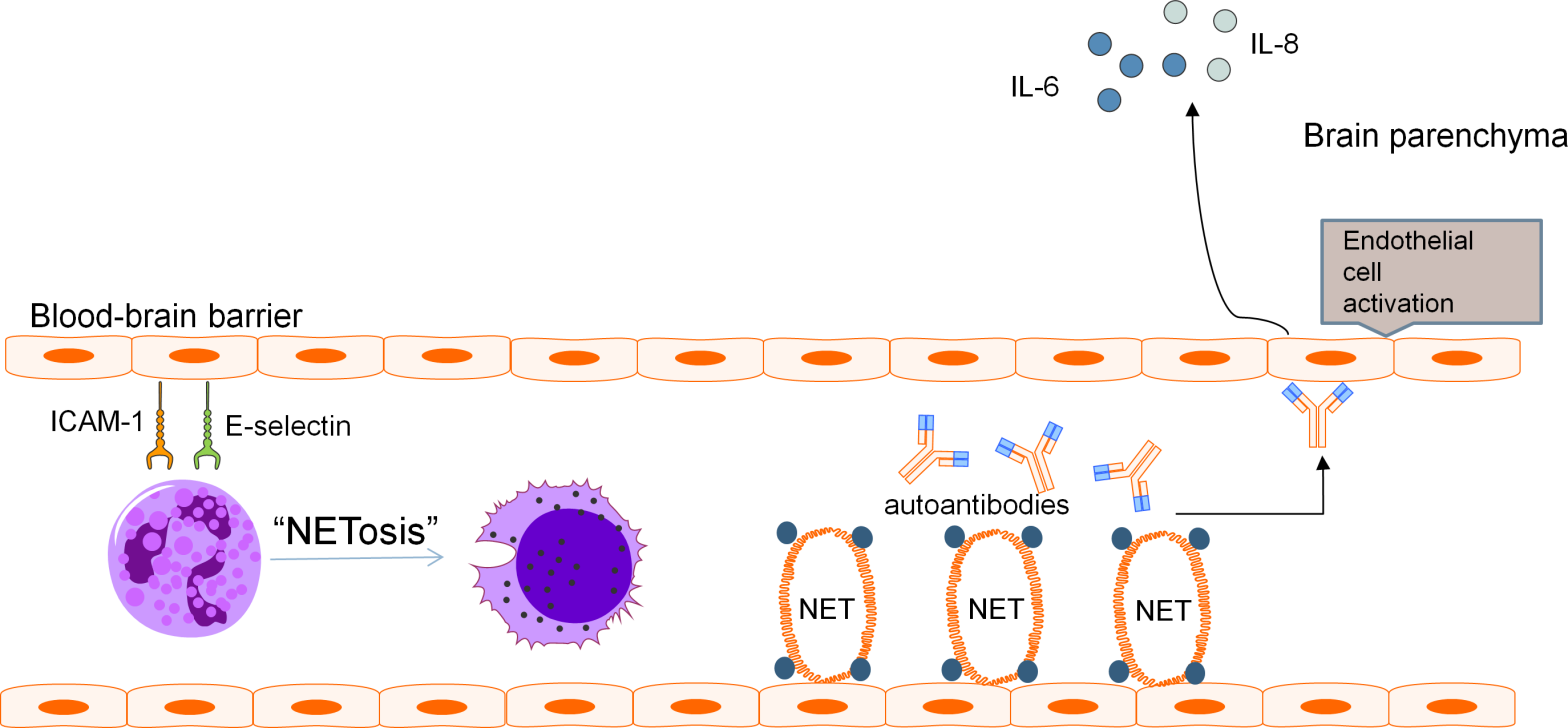
Pathogenic mechanism of BBB disruption. Blood-brain barrier disruption in NPSLE has been found to be related to various distinct but potentially complementary pathways involving neutrophils and their cellular products. Adhesion molecules ICAM-1 and E-selectin present on the vasculature of the brain promote neutrophil arrest and adhesion, leading to recruitment of pathogenic neutrophils to the BBB. These neutrophils produce NETs which cause direct endothelial damage and serve as a source of antigens for the development of autoantibodies including aPL and anti-NR2A/B antibodies. These autoantibodies can further aggravate endothelial damage and promote endothelial activation. The downstream effect is increased permeability of the BBB and secretion of pro-inflammatory cytokines including IL-6 and IL-8.

A two-hit mechanism for the development of NPSLE involving a breach in BBB and neuroinflammation caused by neurotoxic antibodies has been proposed by some authors (86). It is speculated that when BBB dysfunction occurs, the precise microenvironment of the CNS is perturbed, allowing pathologic autoantibodies and inflammatory cells to penetrate, resulting in diffuse NPSLE. Neutrophils are scarce in the CNS under normal conditions as the BBB restricts trafficking of these cells into brain parenchyma. Yet, the infiltration of the CNS by neutrophils in various CNS pathologies is a well-known phenomenon. Evaluation of MRL/lpr lupus mouse model found increased ICAM-1 and E-selectin on vasculature of the brain which preceded development of NPSLE (106). Neutrophils display ligands for these adhesion molecules such as LFA-1 which promote neutrophil arrest and adhesion prior to diapedesis (107). MMP-9-expressing neutrophils have been found to result in severe basal lamina type IV collagen degradation and blood extravasation, promoting BBB breakdown and enabling neuroinflammation (58, 59, 104). Pro-inflammatory cytokines released by netting neutrophils along with activation of the alternative complement pathway induce endothelial-to-mesenchymal transition which causes increased vascular leakage and permeability (47).These mechanisms may contribute to BBB disruption, with subsequent ingress of neutrophils and entry of neurotoxic mediators into the CNS. These effects are further augmented by the increased expression of adhesion molecules on the endothelium of the BBB in SLE which enhances adhesion and subsequent penetration of neutrophils into the CNS, with neutrophil products including MMP-9 and reactive oxygen species compounding BBB dysfunction and permeability to other inflammatory insults (**Figure 1**).

## The interplay between complements and neutrophils in mediating NPSLE

Complement is known to be strongly activated in SLE, resulting in hypocomplementemia being commonly seen in patients with active SLE. In fact, low total complement hemolytic activity and decreased levels of C3 and C4 as many as 90% of patients with diffuse proliferative glomerulonephritis (108). One major mechanism accounting for complement depletion in SLE is the formation of immune complexes which activates complement *via* the classical pathway (108). More recently, the complement system has been found to exhibit a dynamic interplay with neutrophils in the pathogenesis of SLE, the crosstalk facilitated by the presence of complement receptors on the surface of neutrophils including complement receptor 1 (CR1), C3a receptor (C3aR) and C5a receptor 1 (C5aR1) (109). In particular, complement activation has been shown to not only induce phagocytosis in neutrophils but also stimulate NETosis, an interaction possibly crucial in the development of NPSLE (110). Bacteria opsonized with C3b was found to be more potent NETosis inducers than non-opsonized bacteria, suggesting that C3b opsonization *via* the classical or alternative pathways facilitated NET formation (111). The results were corroborated by the finding that the addition of a CR1 antagonist prevented recognition of opsonized microbes by neutrophils, resulting in a decrease in NETosis (111). Similar results were documented from attempts to block C3 receptor *via* iC3b which resulted in marked inhibition of NETosis (112). As previously discussed, NETosis involves the extrusion of intracellular proteins which act as a source of autoantibody formation, and the crosstalk between complement and neutrophils may reinforce this pathogenic pathway in NPSLE.

The complement split product C5a which promotes chemotaxis of neutrophils through the C5a-C5aR1 axis has been found to participate in robust neuroinflammatory reactions. Indeed, factor B knockout MLR/*lpr* mice had decreased neutrophil infiltration into the brain compared to knockout MLR/*lpr* mice (113). The sensitivity of the CNS to effects of C5a is, at least in part, explained by the presence of C5aR1 receptors on the surface of numerous indigenous cell types including microglia, astrocytes and neurons which elicits downstream pro-inflammatory signaling pathways in these cells (114). C5a has been found to exert direct neurotoxic effects through these interactions with cells in the CNS, contributing to cellular apoptosis (115–117). C5a has also been found to contribute to BBB disruption in NPSLE, perturbing the delicate brain microenvironment (118). Signaling through the C5a-C5aR1 axis has been found to activate the NFκB pathway and mediate downstream effects on the expression proteins crucial for the maintenance of the BBB such as tight junction proteins, occludin and zona occludens-1 (ZO-1) (119, 120). The altered expression of these proteins was found to increase BBB permeability in a lupus murine model and an *in vitro* human BBB model (120). The NFκB pathway in endothelial cells also regulate the expression of adhesion molecules such as ICAM-1 and VCAM-1 which mediate the capture and adhesion of leukocytes to the endothelium (121). Increased expression of adhesion molecules on CNS endothelium magnifies the chemotactic properties of C5a on neutrophils and other immune cells, boosting the infiltration of these cells into the CNS through the disrupted BBB.

In a murine lupus model, exposure to pathogenic autoantibodies that bind both DNA and the N-methyl-D-aspartate (NMDA) receptor impaired spatial memory and cognition, inducing NPSLE (122). It was found from C1q knockout mice that dendritic complexity and spine density, but not acute neuronal loss, were better preserved in this model (122). Unlike wild-type mice, C1q-deficient mice behaved normally in an object-place memory task that tests spatial memory (122). In sum, complement proteins and neutrophils have an intricate and dynamic crosstalk which is enabled by the various complement receptors on neutrophils. The mechanisms of complement and neutrophils contributing to NPSLE are similar, and are likely to exist in tandem, amplifying pathogenic effects on the CNS in NPSLE.

## Autoantibodies

Disruption of the BBB enables serum-borne effectors of inflammation to penetrate the CNS, which includes autoantibodies. A hallmark of SLE is the formation of autoantibodies. Some of which are found in the serum, CSF and neuronal tissues and are thought to be directly involved in disease pathways of NPSLE including antiphospholipid (aPL), anti-ribosomal P (anti-P) and anti-N-methyl-D-aspartate (NMDA) receptor subunit NR2A/B antibodies (123–125). Anti-endothelial cell antibodies have been described as having a crucial role in the recruitment of polymorphonuclear leukocytes to foci of inflammation by augmenting cellular adhesion to endothelium (126). Autoantibodies detected in the CSF are the result of either passive transfer of peripherally produced autoantibodies across the BBB or increased intrathecal production (127).

### Anti-N-methyl-D-aspartate receptor subunit NR2A/B antibodies

N-methyl-D-aspartate receptors are a class of L-glutamate receptors that play an important role in cognitive functions including memory and learning and are crucial for spatial memory (128). Antibodies against NMDA receptor NR2A/B subunits (anti-NR2A/B antibodies) are found in the sera of 30-40% of SLE patients and is a convincing candidate as a pathogenic factor in mediating cognitive dysfunction in SLE patients, fulfilling 4 out of 6 stringent pathogenicity criteria for autoantibodies (125, 129).

Affinity purified anti-NR2A/B antibodies have been found to cross-react with double-stranded DNA (dsDNA) (130). *In vitro* and *in vivo* experiments have found that anti-NR2A/B antibodies were capable of inducing apoptotic cell death in neuronal cultures and neuronal damage and loss of hippocampal neurons in a murine model (131, 132). Interestingly, functional NMDA receptors have also been found on the plasma membranes of rat neutrophils after activation (133). Although similar studies of NMDA receptor subunits on human neutrophils have not been reported, it would be a compelling hypothesis for NMDA receptor subsets elaborated on NETs to induce anti-NR2A/B antibodies that recognize both NR2A, NR2B and dsDNA.

In human studies, 57 SLE patients demonstrated an association between serum anti-NR2A/B antibodies and a reduction in short-term memory and depressed mood (134), while another cross-sectional study of 133 SLE patients revealed that serum anti-NR2A/B antibodies displayed impairments in sustained attention, planning abilities and executive function (135). A longitudinal study found an association between increasing titres of serum anti-NR2A/B antibodies with declining working memory function (84). CSF levels of anti-NR2A/B have also been shown to be associated with diffuse NPSLE, especially cognitive deficits (136) and correlate well with SLE disease severity (137). It is worth noting that some studies have not found a positive correlation between serum anti-NR2A/B antibodies and cognitive dysfunction (138). By contrast, CSF anti-NR2A/B antibodies have been consistently shown to be associated with diffuse and central NPSLE (136, 139). The discrepancies of results between CSF and serum anti-NR2A/B antibodies suggest that serum antibodies alone without other factors that disrupt the BBB and increase penetrance into the CNS may not be sufficient to induce neuropsychiatric damage and involvement of SLE (8).

### Anti-phospholipid antibodies

Anti-phospholipid syndrome is characterized by arterial or venous thrombosis, adverse pregnancy outcomes and the positivity of one or more aPL antibodies, including anti-cardiolipin antibodies and anti-β2-glycoprotein 1 antibodies (140). Although this syndrome can occur in isolation, it is more prevalent amongst SLE patients and aPL antibodies are present in up to 30-40% of SLE patients (141). aPL antibodies are known to activate endothelial cells, monocytes and platelets, giving rise a prothrombotic milieu and have been linked with NPSLE (142), suggested by longitudinal studies which showed a correlation between declining cognitive function and persistently elevated aPL levels (143, 144). A study of 51 female SLE patients found a significant relation between persistent anti-cardiolipin antibody positivity with psychomotor speed reduction and decreased conceptual reasoning (143). Another study of 43 SLE patients assessing fronto-subcortical function over 1 decade discovered a significant association between worsening visuospatial functions with hyperlipidemia and positivity of lupus anticoagulant (145).

**Figure 2:**
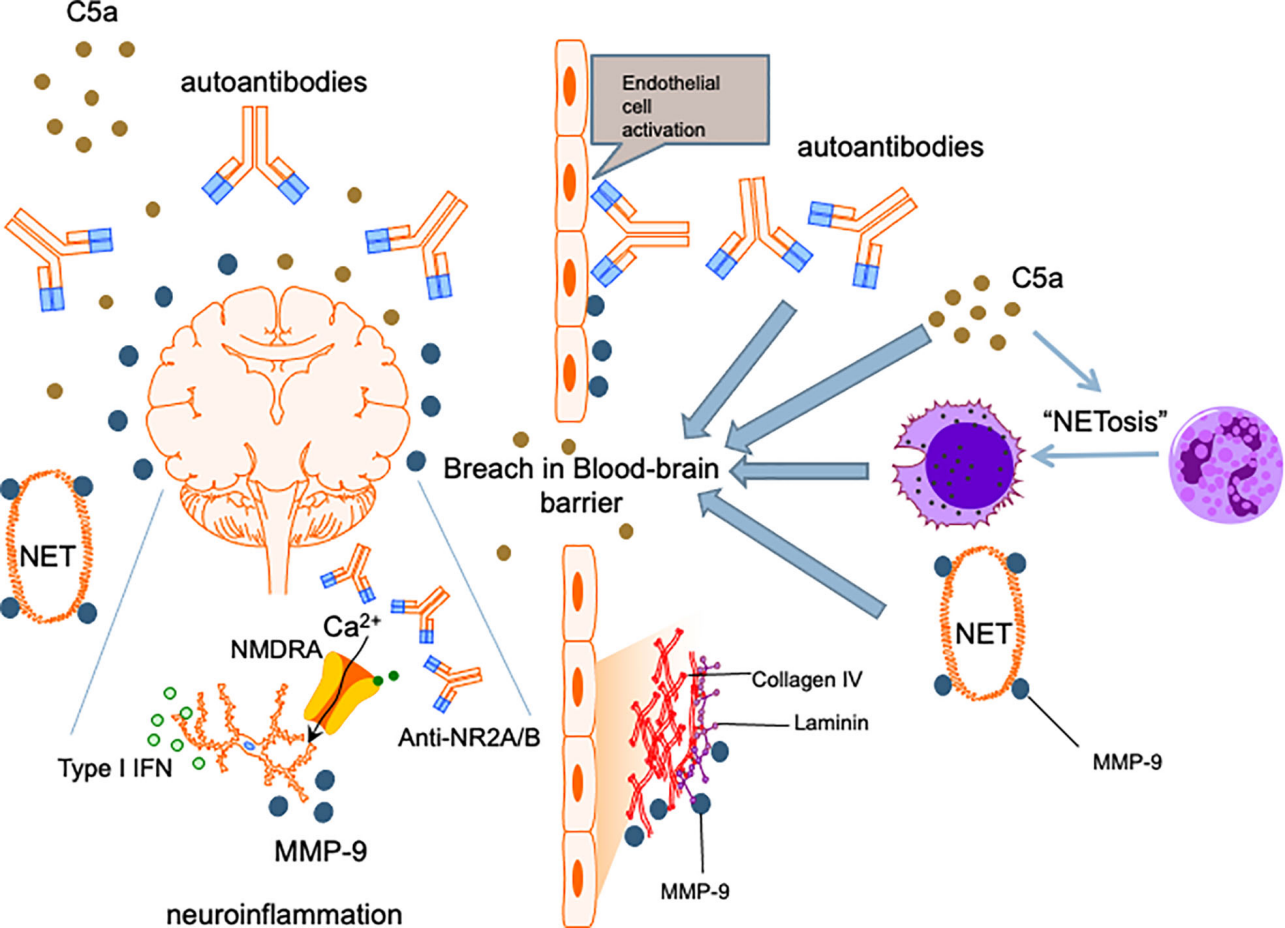
Proposed mechanism for NPSLE. Insults to the BBB allow penetration of the central nervous system by neutrophils and other inflammatory mediators. Cognitive deficits and other neuropsychiatric manifestations are caused by cytokines such as type I IFNs, anti-neuronal autoantibodies including anti-NR2A/B and matrix metalloproteinase-9 instigating neuronal damage and induction of apoptosis. C5a complement fragments are chemotactic and promote migration of immune cells including neutrophils into the CNS, an effect amplified by the ability of C5a to enhance NETosis and increase permeability of the BBB. C5a also exerts direct neurotoxic effects, contributing to further neuropsychiatric insults in NPSLE.

Among patients with SLE, aPL antibody positive patients are about twice as likely to develop NPSLE than patients who were negative (146) and aPL positivity has been long recognized as a strong risk factor for NPSLE development (147). While aPL antibodies accelerate atherosclerosis and cerebral ischaemia (148), aPL antibody positivity has also been linked with NPSLE syndromes that were not necessarily directly related to thrombotic events, such as cognitive dysfunction, chorea and seizures (149, 150). This suggests that aPL antibodies have a pathogenic role in NPSLE beyond their prothrombotic effects. Similar to anti-neuronal antibodies, there have been studies that showed binding of aPL antibodies to neurons and other CNS cells and induction of hyperactive behavior (151), thereby supporting a direct effect of these autoantibodies on the brain parenchyma. Nonetheless, the role of aPL antibodies in NPSLE has been largely ascribed to the sequelae of vascular occlusion and ischaemic events in regions of the brain including the frontal cortex, amygdala and hippocampus (152). Finally, local thrombotic events causing vascular injury is also hypothesized to insult the integrity of the BBB, enabling peripheral effectors including other neuropathic autoantibodies and immune cells to enter the CNS (**Figure 2**) (153). Notably, β2-glycoprotein 1 has been demonstrated on the surface of neutrophils and anti-β2-glycoprotein 1 IgG antibodies can induce NETosis in human neutrophils (154). This will allow this aPL antibody to partake in the neutrophil-related processes mentioned in the preceding sections to mediate NPSLE.

## Neutrophils and neuropsychiatric SLE in a clinical context

Several case reports and a case series described the involvement of neutrophils in the CNS of patients with SLE. For instance, a 14-year-old patient with SLE who presented with loss of consciousness revealed a vasculitic mass with histological features of perivasculitis associated with infiltration of leucocytes including neutrophils (155). CSF pleocytosis with neutrophil predominance was reported in a patient with cerebral lupus presented with acute episodes of haemorrhagic cerebral ischaemia (156). CSF neutrophil pleocytosis was also evident in a 35-year-old SLE patient who was diagnosed to have catastrophic transverse myelitis (157). While the proportion of neutrophils in the CSF of this patient continued to increase (from 76% of 683 white cells/μL to 84% of 1,585 white blood cells/μL) despite empirical antibiotic therapy, treatment with plasmapheresis, intravenous pulse methylprednisolone and cyclophosphamide resulted in a rapid reduction of white blood cell count (to 14 white blood cells/μL) and reversal of white blood cell proportion (from neutrophil predominance to 98% lymphocytes) in the CSF after 3 days, coupled with clinical and radiological improvement of myelitis (157). A 30-year-old patient with SLE who presented with mood and behavioral changes and transverse myelitis was noted to have multiple brain lesions including a large ring-enhancing right basal ganglion lesion on MRI of the brain. Biopsy of the right basal ganglion lesion revealed fibrinoid vascular necrosis and microthrombi with extensive neutrophilic vasculitis and cerebritis (158). Two distinct subtypes of myelitis were later described, comprising of grey and white matter myelitis (159). Patients with grey matter myelitis were more likely to have irreversible paraplegia, monophasic disease course, high SLE disease activity and neutrophilic pleocytosis on CSF analysis (159). In an observational study of 23 patients with SLE that aimed to observe the fluctuation of C3a and C5a in relation to SLE disease flares, in addition to the elevation of serum of C3a and C5a that was more prominent in patients with acute CNS dysfunction compared with patients with active SLE without CNS involvement, postmortem examination of 2 patients revealed neutrophil infiltration that occluded cerebral vessels (160). This observation implied that the release of circulating C3a and C5a may elicit vascular injury and activate neutrophil infiltration, contributing to cerebritis (160). In fact, the critical role of the C5a signaling pathway in mediating NPSLE *via* neutrophil infiltration was evident in an experimental study that demonstrated reduced cerebral neutrophil infiltration, neuronal apoptosis and expressions of p-JNK, pSTAT1 and p-ERK after treatment with C5a receptor antagonist in MRL/lpr lupus-prone mice (161). The potential role of targeting C5a receptor will be discussed further below.

## Neutrophils and neuropsychiatric SLE - the potential mechanistic relationship

Studies that specifically address the pathophysiological impact of neutrophils on clinical neuropsychiatric manifestations of SLE are scant. Clinical situations such as CNS infections and non-steroidal anti-inflammatory drugs (NSAIDs)-induced aseptic meningitis that cause CSF neutrophil pleocytosis may give rise to non-SLE related neuropsychiatric symptoms in patients with SLE (162–165). The use of glucocorticoids (GC) that often leads to peripheral neutrophilia, has been well described to induce a range of neuropsychiatric symptoms reminiscent of NPSLE including anxiety, sleep disturbance and psychosis (166). In patients with hypoalbuminaemia, the lack of circulating GC-binding protein facilitates access of unbound and free GC to the CNS, leading to neuropsychiatric syndromes in patients with SLE (167).

Circumferential evidence that implicates the mechanistic role of neutrophils in mediating NPSLE has been emerging since the 1980s. A study of sera from 53 patients with SLE found an increase in serum neutrophil aggregation, particularly in those with active disease (168). Intriguingly, a high level of neutrophil aggregation activity was prominent in patients with neuropsychiatric symptoms, suggesting the potential role of intravascular leukoaggregates in patient with NPSLE (168). More recently, NGAL (82–84) and NETs (169) in relation to NPSLE have been described although their roles in NPSLE require further elucidation and validation.

NETs have been detected in a number of CNS diseases including acute thrombotic strokes, particularly that NETs appear to promote coagulation and thrombosis (169). NETs were also observed to be involved in brain parenchymal oedema of traumatic brain injury in mice and gliomas (169). While a SLE patient with non-aPL antibody-mediated Libman-Sacks endocarditis who presented with seizures and psychosis was found to have active NETs formation in the mitral valve and peripheral circulation, no NETs remnant or heighted elastase activity was detected in the CSF (170). More observations and experiments are required to assess if NETs contribute a role to the pathogenesis of NPSLE in lupus patients.

## Targeting neutrophils in NPSLE

Increasingly, there has been interest in targeting the neutrophil axis for therapeutic application in NPSLE. Inhibition of serine protease neutrophil elastase in a mouse model of SLE revealed reduced NETosis, lowered levels of some autoantibodies and conferred protection against arterial and venous thrombosis (171). Another murine study investigated the use of Cl-amidine, an inhibitor of peptidylarginine deiminases (PAD) which is an enzyme crucial in the formation of NETs (172). The study found that Cl-amidine led to robust improvements in vasculogenesis, endothelial function and thrombotic risk.

It is pertinent to remember that while NET formation functions as part of the innate immune system against pathogens, while excessive NETosis is implicated in the pathogenesis of SLE and other autoimmune and inflammatory disorders. Indeed, inhibition to or knock-out of PAD has been demonstrated to reduce the severity of autoimmune disease, degree of inflammation and production of autoantibodies through a mechanism dependent on inhibition of NET formation (173). Furthermore, PAD inhibition is also associated with a decrease in T-helper cell inflammatory responses mediated by the Th1 and Th17 subtypes (174–176). Given the intricate balance between maintaining immune homeostasis and autoimmunity, inhibition of NETosis may potentially impair protective host immune responses (177). PAD-deficient mice have been found to be more susceptible to bacterial infections than healthy mice (178). Another study reported that PAD-knock-out mice developed systemic inflammation and bacterial keratitis (179). Cyclosporine is an immunosuppressive agent which can inhibit NETosis through inhibition of calcium flux (180). It is well known that the use of immunosuppressive drugs such as cyclosporine is associated with infections (181), contributed in part by an inhibition of neutrophil-related pathways. Hence, inhibition of NET formation has to be undertaken with caution since they may predispose patients to potentially deleterious infective complications.

Neutrophils bear receptors such as C5a receptor that enable them to coordinate their effector functions with other elements of the innate immune system including the complements (182). Avacopan is an orally administered small-molecule C5a receptor antagonist which selectively blocks stimulatory effects of C5a, namely neutrophil chemoattraction and activation (183). A clinical trial on patients with ANCA-associated vasculitis found that avacopan was comparable to tapering doses of prednisone with respect to remission at week 26 but with fewer glucocorticoid-induced toxic effects (183). In the same trial, avacopan was also shown to confer renal protective effects as evidenced by improvements in renal function and albuminuria which are corroborated by effects seen in other human trials and murine studies (184–186). The potent renal protective pharmacological action of avacopan is attributed to the blockade of the C5a-C5a receptor axis which arrests chemoattraction and activation of neutrophils in glomeruli (184, 187). While clinical studies using avacopan in the treatment of SLE have not been performed, we speculate that this medication would be efficacious in neutrophil-mediated lupus manifestations in the CNS. Complement activation has also been implicated in antiphospholidpid syndrome (188). Eculizumab inhibits the cleavage of C5 to C5a and C5b, interrupting the complement mediated proinflammatory and prothrombotic environment (189). Indeed, eculizumab has been used in refractory catastrophic antiphospholipid syndrome (189). It has been proposed that blocking C5 activation and inhibiting C5aR1 signaling would limit the inflammatory damage mediated by neutrophils (190). Microfluidic studies have demonstrated that both avacopan and eculizumab inhibit both neutrophil chemotaxis and swarming functions (190). However, avacopan but not eculizumab preserves neutrophil phagocytosis of *S. aureus* bioparticles (190). Considering the known complication of increased susceptibility to encapsulated bacteria by eculizumab due to the inhibition of the distal complement components, avacopan appears to have better safety profile compared to eculizumab (183).

## Conclusion

Neutrophils have been recognized as instigators of autoimmunity and effectors of tissue damage in SLE, therefore it is plausible that inhibition of neutrophil effector functions can be investigated as a potential therapeutic strategy for NPSLE. Breach of the BBB, neutrophil activation, transmigration and subsequent intrathecal and peripheral release of NETs are thought to act in concert with neuropathogenic autoantibodies including anti-NR2A/B antibodies to cause inflammation and neuronal cell death, leading to neuropsychiatric manifestations of SLE (191). LDNs represent a unique subset of neutrophils in SLE and their production of proinflammatory cytokines and other signaling molecules may have a role in orchestrating neurotoxic insults and other end-organ damage in SLE (192). Cellular products of LDNs such as MMP-9 and NGAL also play an instrumental role in disrupting the BBB and causing neuroinflammation in NPSLE. The diversity of clinical phenotypes and features of NPSLE is reflection that NP syndromes are a complex facet of SLE which involves distinct yet interwoven pathways and mechanisms. Given the central role that neutrophils play in the pathogenesis of NPSLE, therapeutic agents targeting the neutrophil axis warrant investigation for they could hold the key to the treatment and even prevention of NPSLE.

## Author contributions

Conceptualization, TS and ST. Writing—original draft preparation, TS, AM, and ST. Writing—review and editing, TS, AM, and ST. Supervision, AM and ST. All authors contributed to the article and approved the submitted version.

## Conflict of interest

The authors declare that the research was conducted in the absence of any commercial or financial relationships that could be construed as a potential conflict of interest.

## Publisher’s note

All claims expressed in this article are solely those of the authors and do not necessarily represent those of their affiliated organizations, or those of the publisher, the editors and the reviewers. Any product that may be evaluated in this article, or claim that may be made by its manufacturer, is not guaranteed or endorsed by the publisher.
